# Historic Cities: Issues of Urban Conservation, edited by Jeff Cody and Francesco Siravo. Getty Conservation Institute, Los Angeles, 2019. 610 pp. $60.00. ISBN9781606065938

**DOI:** 10.1186/s43238-021-00022-0

**Published:** 2021-03-12

**Authors:** Earl Kessler

**Affiliations:** Bureau of Humanitarian Assistance, USAID, 555 12th Street, NW, Washington, DC 20004 USA


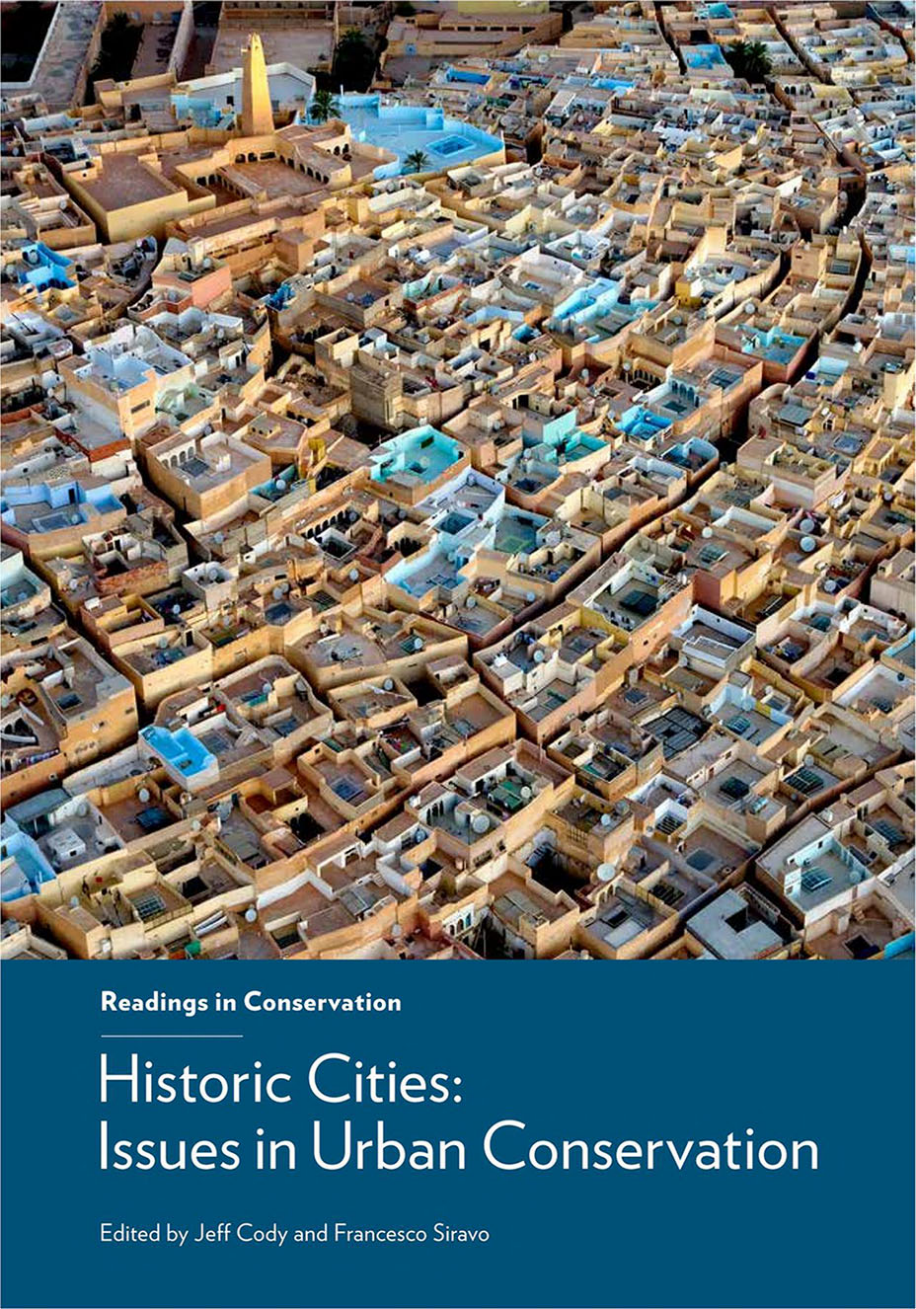


Jeff Cody and Francesco Siravo have captured the crisis confronting historic cities and issued a call to action in *Historic Cities: Issues of Urban Conservation*. The book consolidates thinking about, investigations into and actions taken that embarks us on a journey to educate us about and rescue historic cities for the value they represent to us now and into the future. The world is now URBAN and as much as the Development Set still romanticizes rural development at the expense of urban areas, it is cities—all sizes and kinds of cities—that are the future.

The book is divided into eight thematic parts preceded by an introduction from the editors, and concluded with illustrative images in the shape of visual summaries. These different sections address the changing nature of historic cities, their geographical diversity, the transformation of traditional cities, how to read the ciy. They address the issues of continuity, identifying heritage values of cities, the challenge of sustainable urban conservation as managing historic cities. For each part, the editors have selected a range of key authors from the early 19th century to 2017, from different professional backgrounds, and regions of the world to offer as wide as possible a panorama. The format allows the reader to take in slowly the sequence of chosen selections. The selections track and illuminate thinking that is bringing change to how we consider historic cities and integrate them into the future. The short bursts of input allow the reader to digest what is presented and the cumulative effect brings home a clarity of why and what has to happen now to make historic cities the resource they are.

From the outset we are presented, through variegated sources, disciplines and perspectives with clear admonitions to not ignore the multiple values and purposes historic cities hold. Most importantly historic cities reflect and provide backdrop to our humanity if only we would look. The book calls out the human aspects pushed aside in discussions of a new and different value system that threatens historic cities—the market value of land and the restive impacts of societies in change. From the very first page we are told ‘the historic city demands that we understand the profound disconnect that occurred in city building since the industrial revolution … we need to recognize and appreciate the subtle clues that set historic places apart from the look-alike sameness of most present-day urban developments.’ (p. 1)

The book points out that the world economy since the industrial revolution has changed the rules of the game. The slow evolution that created cities historically has been taken over by a different set of values and pace of development. Historic cities have become commodities and it is land value and space for new-world toys—cars—that rule and despoils the humanity of places relegated to a footnote by new-world developers and politicians. There is more to cities and neighborhoods than returns on investment.

The sense of loss that permeates the book is palpable in the first parts. It calls on those working in the field to balance and move from theory to practice. One of the more poignant expressions of that loss exposes the impacts of the quest for misguided ‘modernity’ and the mass-tourism made possible by prosperity that has made frivolous places once regal and sacred. Salvatore Settis in his selection ‘If Venice Dies’ cuts no slack in his assessment of what has happened in Venice. Seyyed Hossein Nasr forcefully states ‘Euro-centrism also sees its view of the world as the only worldview … Not even Genghis Khan could do what we are doing through “development” … There is nothing more important than to realize that what remains of our historic cities, of our sacred sites and sacred places, is significant not only for national identity or archeological records but also for our own identity as human beings.’ (p. 20–21)

This book records the results of the wonton destruction of exceptional places destroyed in the name of Modernism and economic development. The book puts into perspective and helps explain this reviewer’s personal sense of loss, and perhaps that of others who may read it, who have been witness to the destruction of wondrous places and their cultures as a result of the overriding forces of change that Cody and Siravo explain. Examples abound but the two that won’t leave me alone are St. Louis, Missouri where I grew up and studied architecture and Cartagena, Colombia where I was a Peace Corps Volunteer in the 1960’s. In St. Louis, it was to see one of, if not THE best, Victorian housing stock knocked down to be replaced by the then award winning Pruitt-Igoe project. The other is Cartagena, Colombia overtaken by tourism. Pruitt-Igoe in St. Louis was designed for the urban poor that became the most dangerous place to live in the city and was ultimately dynamited in a huge implosion to remove the entire development from existence. It forced the residents, who never made the transition to high-rise living, to disburse and left a gaping wound in the fabric of the city that to this day has never quite healed. The ‘reason’ was the new modernity of Corbu’s Garden City that only masked the racism behind the decisions to rebuild downtown St. Louis.

Cartagena, Colombia painfully displays what the selections emphatically lament - the danger of mass-tourism and its impact on what was once one of my favorite places on earth. The 16th century city was the gateway to exporting the gold plundered from the societies the Spanish conquered as well as the gateway of slavery into the Andean region. Once Cartagena *La Heroica*, the Heroic, for its defense against Vernon the pirate among others; it is now Cartagena *La Turistica*, and that is not an improvement. It lost its soul as ‘economic’ pressures drove out the people and destroyed the lively, inclusive central market that really gave the city its flavor to make room for the International Convention Center, surrounded by a determined fence to keep the ‘*Cartageneros*’ out with a design not unlike the Kunsthaus blobstyle, the Friendly Alien—or not so friendly—in Graz Austria. Settis clearly states, ‘Citizens—and not tourists—are the real life blood of the city.’ (p. 189) Poor Venice! Poor Barcelona! Poor Cartagena!

The book provides through a diverse, inclusive chorus of authors a new way of thinking about historic cities that changes the static concept of conservation as monuments to a dynamic one that requires the rethinking of historic cities as living resources for future urban growth. Local context is key. Senam Okudzeto, provides an African voice that reminds us that ‘anti-colonialist movements identified Ghanaian urban heritage very closely with social uses of space and on going cultural traditions. Historic structures, including recent ones, are the physical vessels in which social traditions can survive.’ (p. 436–437)

Non Arkaraprasertkul and Matthew Williams reflect on the irony of the events in Shanghai, that now, after wonton destruction, ‘regards historic preservation of selected sites, including the traditional alleyways of Shanghai, as essential to the branding of a city with global ambitions.’ (p. 444) The book highlights that the destruction in Shanghai of the alleyway houses, not unlike the destruction of the shop houses in Singapore only to be rebuilt ‘modern,’ is mirrored in the selections on urban renewal of the 1950’s and 60’s in the USA. Gratz with Mintz build on Jane Jacobs’ struggle against car-induced demolitions, slum clearances and urban renewal projects from the 1930s to the 1970s. Cederna offers a glimpse of positive thinking ‘We must fix in our minds on the conviction that integral preservation of the old and creation of the new in cities are complementary tasks … That ancient and modern have distinct material and spiritual prerogatives and are mutually necessary.’ (p. 10)

What needs to be made clear is that for all the good that the book points out that historic cities can contribute to the quality of life of the people of a city, it is and will be the political will of those that govern, that foment ‘development’, that provide the guidance a local culture values, and that invests in the maintenance of historic cities that will make the difference. The battle is on and is far from over. The latest assault that misses the point altogether is the reverence for technology and the new systems that would have us believe that they are an improvement in our lives. Shannon Mattern shatters that notion stating clearly ‘The city is not a computer and technologists and political actors speak as if they could reduce urban planning to algorithms’. (p. 575) ‘Smart Cities’ are but a distraction and were they Smart the future of historic cities, as this well conceived book militates for, would be bright.

In sum, the book’s length and/or cost might be daunting for some readers, but that it is well worth the effort to take the time to savor the prose, learn from the graphic examples, and determine how to take the lessons implied by the book and apply them to actual challenges. In our post-Covid world, with so many unknown changes to come in historic cities worldwide, this book’s importance and significance is even more critical than it was, before Covid-19 came into our lives, when Cody and Siravo assembled such a stimulating cluster of readings, comments on those readings, and images that complement them.

## Data Availability

Not Applicable.

